# The Lepidoptera of White Sands National Monument, Otero County, New Mexico, USA 9. A new species of *Givira* Walker (Cossidae, Hypoptinae) dedicated to Delinda Mix, including a list of species of Cossidae recorded from the Monument

**DOI:** 10.3897/zookeys.655.11339

**Published:** 2017-02-13

**Authors:** Eric H. Metzler

**Affiliations:** 1 Adjunct Curator of Lepidoptera Michigan State University; Research Collaborator National Museum of Natural History, Smithsonian Institution; Research Associate Museum of Southwestern Biology the University of New Mexico. Research Associate McGuire Center for Lepidoptera and Biodiversity, University of Florida, P.O. Box 45, Alamogordo, NM 88311-0045 USA

**Keywords:** Endemism, evolution, U.S. National Park Service, U.S. Army, White Sands Missile Range, Tularosa Basin, biological diversity, white gypsum dunes

## Abstract

The U.S. National Park Service initiated a 10-year study of the Lepidoptera at White Sands National Monument, Otero County, New Mexico in late 2006. *Givira delindae* **sp. n.**, discovered in 2007 during the first year of study, is described here. The male and female adult moths and genitalia are illustrated. The name is dedicated to Delinda Mix, mother of Steve Mix. The species of Cossidae recorded from the Monument during the study are listed.

## Introduction

The purpose of this paper is to describe a new species of *Givira* Walker (Cossidae) from White Sands National Monument. In 2006 White Sands National Monument invited me to conduct a 10-year study of moths at the Monument with the purposes to compile an inventory of moths, and describe new species in habitats within and immediately adjacent to the white gypsum dunes in the Monument. The White Sands National Monument protects 284.9 km2 (110 square miles), about 40%, of the world’s largest snow-white gypsum dune field. The remainder of the 275 square miles formation is under the jurisdiction of the U.S. Army’s White Sands Missile Range. The formation is located in the northern Chihuahuan Desert in southern New Mexico’s Tularosa Basin (Schneider-Hector 1993).

The Western National Parks Association (WNPA) in Tucson, Arizona is a nonprofit 501(c) (3) education partner of the National Park Service that supports 71 national park partners across the West, by developing products, services, and programs that enrich the visitor experience. WNPA provided considerable moral support and renewable grants of $7,500 per year during the first three years of my study at White Sands National Monument. I decided to assist WNPA in a fund raising event by agreeing to name a new species of moth, as directed by the winner of an auction conducted by WNPA. The auction, with approval of the National Park Service, was conducted on the popular web-site www.ebay.com. Steve Mix submitted the winning bid, and he chose to have the moth named after his mother because of the lasting nature of this naming opportunity. I received no remuneration in this fund raising venture, and by volunteering my personal money, time, expertise, and experience I was able to help WNPA gain world-wide positive publicity while raising some much needed cash. The rewards to me were being able to help WNPA and Steve Mix honor his mother, which is just so very sentimental.

Prior to this study 20 species of moths were recorded from the Monument (Stroud 1950). None of Stroud’s reported species is unusual for the Tularosa Basin. The lack of lepidopteran specimens until my study can probably be attributed to the dearth of insect collecting in the gypsum dunes ecosystem in New Mexico because the dunes were private property and are now under the control of the U.S. National Park Service and the U.S. Army. In the period 9 February 2007 through 30 July 2016, I collected more than 600 named species (unpublished data) of Lepidoptera from the Monument plus approximately 40 undescribed species of moths. This is the 13th description of a new species of moth emanating from the study (see Metzler 2014, 2016; Metzler et al. 2009; Metzler and Forbes 2011a, 2011b, 2012; Metzler and Landry 2016, Metzler and Lightfoot 2014, Wright 2012, 2014, Wright and Gilligan 2015).

## Materials and methods

Moths and other night flying insects for this study were collected in U.S.D.A. type black-light traps, as described in Smith et al. (1974), or at black light and sheet as illustrated in Covell (1984). Samples were taken in diverse habitats within the dunes and the adjacent desert habitats in White Sands National Monument. I assigned a unique code, i.e. WSNM 1, WSNM 2, etc. through WSNM Z to each sample site. The date/locality label of each specimen includes the site code along with the latitude, longitude, elevation, and a one or two word description of the habitat at each site. All except easily identified species of moths (e.g. *Hyles lineata* (Fabricius), Sphingidae), were retained, sorted to species, and selected specimens were spread and labeled. All non-lepidopteran insects from the traps were placed in 95% ethanol and deposited in the Museum of Southwestern Biology at the University of New Mexico, Albuquerque, New Mexico.

The genitalia were examined by generally following procedures outlined in Clarke (1941), Hardwick (1950), Lafontaine (2004), and Pogue (2002). Abdomens were removed from the moths, dipped in 95% ethyl alcohol, and soaked in 10% KOH for up to 30 minutes at 50°C. Genitalia were dissected in 25% propanol. Genitalia were stained with Orcein in propanol. The genital organs were dehydrated in 100% propanol, and slide mounted in Euparal.

Terminology for regions of the wing and wing markings comes from Mikkola et al. (2009) and genital structures from Klots (1970). Terminology for color comes from Jewell and Abate (2001). Forewing lengths were measured to the nearest 0.1 mm, from the base to the apex excluding fringe, using a Leica MZ 12 stereo-microscope with a Wild Schraubenmikrometer okular 15× SK.

The photographs of the adults of the types of *Givira carla* and *Givira durangona* illustrated in this paper were taken and processed by Karolyn Darrow and made available by Patricia Gentili-Poole. The photographs of the adults of *Givira delindae* sp. n. were taken with a Nikon D7100 equipped with an AF-S Micro Nikkor 105mm 1.28 GED VR lens and a small homemade light-box, of 4” diameter × 4” long white PCV pipe, illuminated with a 60 LED ring light. The photographs of the genitalia were taken with a Nikon D7100 mounted on a Leitz Aristophot with an 8 cm Summar and an 80 mm condenser. The images the adults of *Givira delindae* and the genitalia were processed with Zerene Systems software and Photoshop CS6 software.

Specimens of Lepidoptera cited in this paper are deposited in the following collections:

EHM
 Eric H. Metzler for subsequent transfer to MSUC 


MSUC
 Michigan State University Albert J. Cook Arthropod Research Collection 


NMSU
 New Mexico State University Arthropod Collection 


UNM
 University of New Mexico’s Museum of Southwestern Biology 


USNM
 National Museum of Natural History, Smithsonian Institution 


## Taxonomy and morphology

The North American species of the family Cossidae were revised by Barnes and McDunnough (1911) wherein they refined the definition of the genus *Givira* and included 11 species. The Barnes and McDunnough revision of 1911 was updated by Dyar and Schaus (1937) when all species of *Givira* from the New World were included. When Hodges (1983) updated the list of *Givira* for North American, the number of species was 13.

Most of the North American species listed in the genus of *Givira* are dark colored or have substantial dark smudges on the forewing, i.e. *Givira anna* (Dyar, 1898), *Givira arbeloides* (Dyar, 1899), *Givira cleopatra* Barnes & McDunnough, 1912, *Givira ethela* (Neumoegen & Dyar, 1893), *Givira francesca* (Dyar, 1909), *Givira lotta* Barnes & McDunnough, 1910, *Givira lucretia* (Barnes & McDunnough, 1913), *Givira marga* Barnes & McDunnough, 1910, *Givira minuta* Barnes & McDunnough, 1910, *Givira mucida* (Hy Edwards, 1882), and *Givira theodori* (Dyar, 1893). In contrast *Givira carla* Dyar, 1923, *Givira cornelia* (Neumoegen & Dyar, 1893), *Givira durangona* (Schaus, 1901), and *Givira delindae* sp. n. are substantially white with few or no dark markings.

Barnes and McDunnough (1911) relied on wing venation and the habitus of the adults to define genera and species. Dalle-Torre (1923) published confused taxonomy by making generic and subfamily transfers without explanation. His combinations were corrected by later authors (e.g. Dyar and Schaus 1937). Dyar and Schaus (1937) clarified the Barnes and McDunnough (1911) definition of *Givira* in order to account for species from Latin America. Clench (1957) modified the taxonomy to accommodate nomenclature of Latin American and Old World species. For the Neotropics, Donahue (1995) listed 86 species of *Givira* including nine species occurring in the southwestern United States.

Old World treatments of Cossidae Daniel (1956, 1958, 1960, 1962, 1964, 1965) and Zagulyaev (1978) placed some emphasis on the morphology of individual antennal segments, whereas Borth et al. (2011), Hua et al. (1990), Hua (2001 (2002)), Jimbo (2011), Roepke (1957), Saldaitis and Ivinskis (2010a, 2010b), Wiltshire (1982), Yakovlev (e.g. 2008a, 2008b, 2011a, 2011b, 2011c, 2015a, 2015b), Yakovlev et al. (2013, 2015), Yakovlev and Saldaitis (2008), and Yakovlev and Witt (2015, 2016) emphasize adult habitus and genitalia without illustration of individual antennal segments. Neither Schoorl’s (1990) nor Edwards et al.’s (1998) reviews of Cossidae classification employed antennal morphology. More recent descriptions of *Givira* from the Western Hemisphere, (Clench 1956 (1957), 1957, Ureta 1957, and Zukowsky 1954) do not refer to antennal segments. I do not refer to individual antennal segments for three reasons: 1) only Old World treatments illustrated antennal segments; 2) because the *Givira*-*Langsdorfia* (see Clench 1957, page 132) group of genera, occur only in the New World, no illustrations of antennal segments of *Givira* or other species in the group are available for comparison; and 3) illustrations of antennal segments are not being used in modern works, including Yakovlev’s many recent descriptive publications.

## Results

### Givira delindae

Taxon classificationAnimaliaLepidopteraCossidae

Metzler sp. n.

http://zoobank.org/F4D84641-CB30-45FA-B0BA-CACABF97FE96

Figs 1–4 , 9 , 12 , 13–15

#### Type material.

**Holotype** ♂, pinned with labels as follows: “USA: N[ew]M[exico]: Otero Co., White Sands Nat[ional] Mon[ument], interdune vegetation, 32°46.69'N 106°11.38'W, 4,000’, 10 August 2010, WSNM 8, Eric H. Metzler uv trp, Accss # WHSA 00131, USNMENT 00913976, HOLOTYPE *Givira delindae* Metzler 2017 [USNM]. **Allotype** ♀, pinned with labels as follows: “USA: N[ew]M[exico]: Otero Co., White Sands Nat[ional] Mon[ument], interdune vegetation, 32°46.42'N 106°10.51'W, 4,012’, 4 June 2016, WSNM Z, Eric H. Metzler uv trp, Accss # WHSA 00131, Allotype *Givira delindae* Metzler 2017 [USNM]. **Paratypes**: 104 ♂, 3♀ All paratypes are “USA: N[ew]M[exico]: Otero County: White Sands Nat[ional] Mon[ument], Accsn#: WSNM-00131.” The specimens with discrete sample sites are “Eric H. Metzler uv trp” Sample sites within the dunes are: WSNM 1, open dunes, no vegetation, 32°45.78'N, 106°11.39’ W 4,014,’ 13 May 2007, (1♂), WSNM 2, interdunal vegetation, 32°45.57'N, 106°11.59'W, 4,006,’ 13 May 2007 (3 ♂, 1 ♂ gen. on slide USNM 127,559), WSNM 3, edge of dunes/basin, 32°45.70'N, 106°11.24'W, 4,001’ 13 May 2007 (4 ♂, 1 ♂ gen. on slide USNM 127,555), WSNM 8, interdune vegetation, 32°45.685'N, 106°11.379’ W, 4,000’ 3 June 2008 (3 ♂), 22 July 2008 (2 ♂), 20 June 2009 (1 ♂), 8 September 2015 (1♂), WSNM 9, interdune vegetation, 32°45.724'N, 106°11.315'W, 4,000’ 3 June 2008 (2 ♂), 22 July 2008 (2 ♂), 10 June 2009 (1♂), 20 June 2009 (2 ♂), 10 June 2013 (2 ♂) WSNM B, interdunal vegetation, 32°45.596'N, 106°11.494'W, 4,000’ 3 June 2008 (6 ♂), WSNM C, crest of dunes near vegetation, 32°45.668'N, 106°11.418'W, 4,014’ 3 June 2008 (2 ♂), 10 August 2010 (4 ♂), WSNM D, interdunal veg., 32°46.620'N, 106°10.824'W, 4,008’ 19 May 2009 (2 ♂), 10 August 2010 (4 ♂), WSNM F, interdune vegetation, 106°10.838'W, 32°46.643'N, 4,008’ 19 May 2009 (1 ♂, 1♀ genitalia on slide USNM 127,563), 10 August 2010 (1♂), 10 June 2013 (1♂), 19 May 2015 (3 ♂), 20 May 2015 (2 ♂), 5 Sept 2013 (3 ♂), 4 June 2016 (3♂), WSNM Z, interdune vegetation, 32°46’42.4"N, 106°10'51.55"W, 4,012’ 4 May 2016 (1♂), 4 June 2016 (1♂), 5 June 2016 (3 ♂, 1 ♂ genitalia on slide USNM 127,556), 6 June 2016 (3 ♂, 1♀genitalia on slide USNM 127,557), 7 June 2016 (4 ♂, 1 ♂ genitalia on slide USNM 127,560). 10 June 2016 (5 ♂), 13 June 2016 (6 ♂, 1♀, ♀ genitalia on slide USNM 127,558), The next 20 specimens were collected by Greg Forbes: vicinity of Admin. Building, 32°46'46.60"N 106°10'26.70"W, 4006’, 14 May 2009 (1♀, genitalia on slide E.H.M.721), Interdunes at W end Big Pedestal Rd. 2.5 mi SW Admin. Bldg. (= terminus Big Pedestal Rd.), 32°45’31.76"N 106°11'34.20"W, 4006’, 21 June 2007 (1♂). 22 June 2007 (1 ♂), 15 August 2007 (1♂), 11-12 May 2008 (1♂), 30 May 2008 (1♂), 11 June 2008 (2♂), 6 July 2008 (3♂), 17 July 2008 (1♂), 6 August 2008 (1♂, wings on slide E.H.M.726, hind leg on slide E.H.M.727). Ca. 100 m NE terminus Big Pedestal Rd. 22 June 2007 (1♂), 30 May 2008 (2♂), Storage area (= boneyard) 32°46'43.12"N 106°10'48.86"W, 4006’, 30 May 2008 (3♂), 6 July 2008 (1♂), 14 May 2009 (1♂ genitalia on slide E.H.M.713), Thirty specimens, all from within the dunes of White Sands National Monument were excluded from the type series because of poor conditions of the wings.

#### Etymology.

The specific name of this species, *delindae*, a noun in the genitive case, honors Delinda Mix for the support and encouragement she gave to her son, Steve Mix, who was interested in studying butterflies and moths as a young man. He maintains his interest in Lepidoptera.

#### Diagnosis.

**Figures 1–8.**
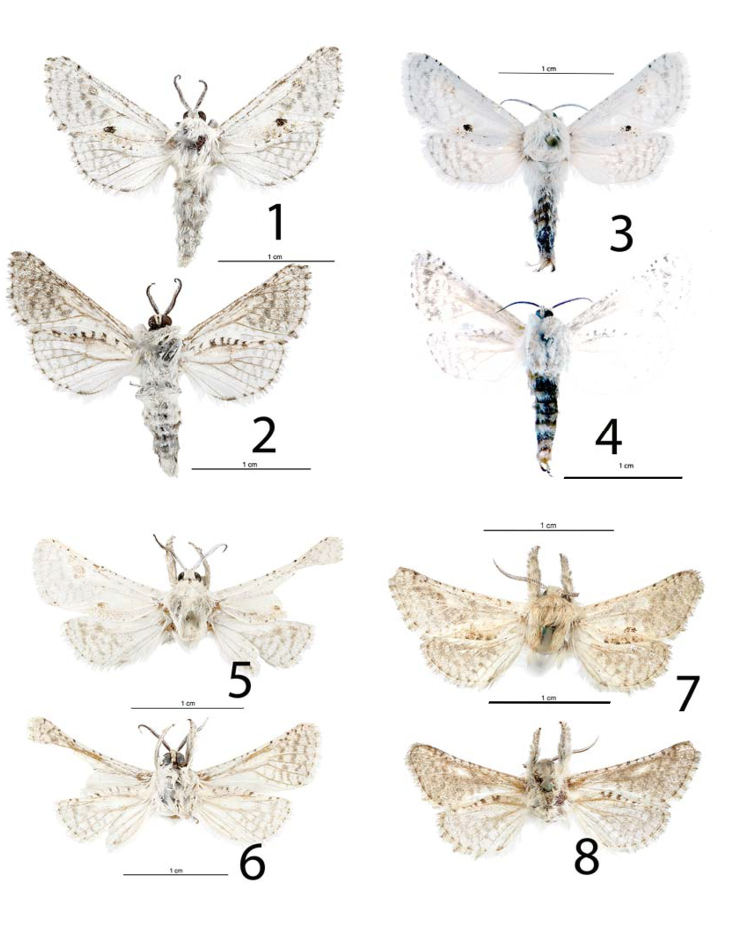
*Givira* adults. **1** *Givira delindae* Holotype ♂ upperside **2** *Givira delindae* Holotype ♂ underside **3** *Givira delindae* Allotype ♀ upperside **4** *Givira delindae* Allotype ♀ underside **5** *Givira carla* Holotype ♂ (photographed after dissection) upperside **6** *Givira carla* Holotype ♂ underside **7** *Givira durangona* Holotype ♂ (photographed after dissection) upperside **8** *Givira durangona* Holotype ♂ underside.

**Figures 9–12.**
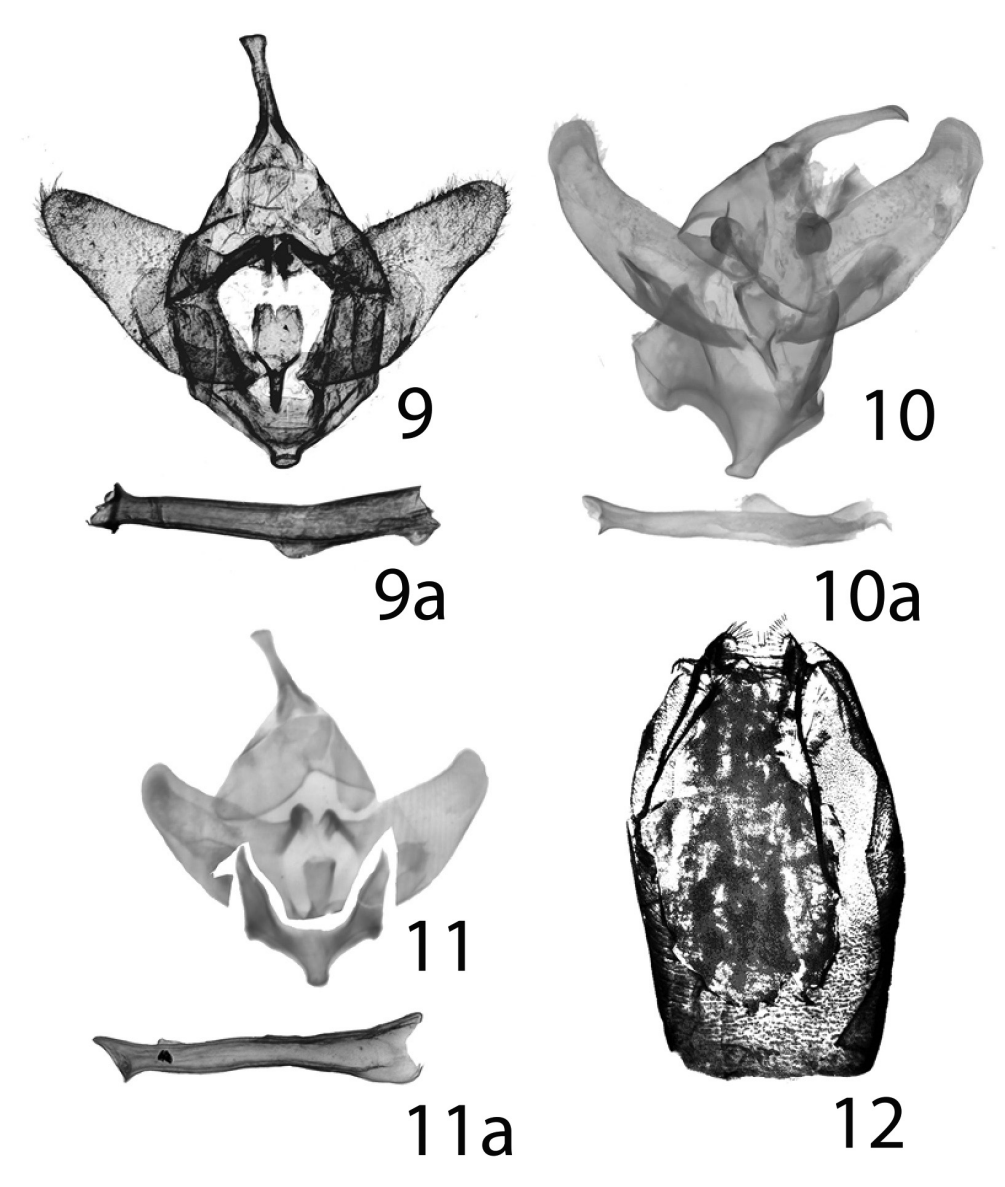
*Givira* genitalia **9** *Givira delindae* ♂ Paratype USNM slide 127,556 **9a** (genital capsule) **9b** (aedeagus) **10** *Givira carla* Holotype ♂ USNM slide 85,292 **10a** (genital capsule) **10b** (aedeagus) **11** *Givira durangona* Holotype ♂ USNM slide 85,295 **11a** (genital capsule) **11b** (aedeagus) **12** *Givira delindae* ♀ genitalia Paratype USNM slide 127,563.

The diagnostic features are the satiny-white wings with vague pale-gray markings, a small contrasting dark patch of scales near the middle, closer to the dorsal margin, of the forewing (Figs 1, 3, 15), The thorax and abdomen are velvety white. Abdominal tufts have gray-tipped scales. The posterior dorsal margin of the thorax has prominent semi-erect tufts of scales, the abdomen has two basal erect dorsal tufts of gray-tipped scales, and the posterior end of the abdomen has a prominent often semi-erect, furcate tuft of scales. In addition to the contrasting dark patches on the forewings, there are two tufts of semi-erect scales along the path of the postmedial line. The other fore wing markings are more or less contrasting three faint-gray lines, parallel to the outer margin in the postmedial area. The costa of the forewing of *Givira delindae* may or may not have one, two, or three rows of costal and sub-costal tiny black spots. The markings of the hindwing are a series of parallel gray widened lines that are more conspicuous at the costal margin. Worn specimens are dull white shaded with gray, and most pinned specimens are greased and oily-gray in appearance. The abdomen is full of fatty tissue (obvious when dissected) hence the reason most pinned specimens are greasy. The forewings and hindwings of *Givira carla* are white, without dark smudges and with three or four faint obscure pale-gray shades parallel to the outer margin (Fig. 5). The forewing of *Givira carla* is not satiny white and has numerous small black spots. The post medial dark markings are brown on *Givira cornelia*, and when compared to *Givira delindae*, the postmedial line markings of *Givira cornelia* are longer, when measured from near the tornal angle towards the costa. The wings of *Givira cornelia* are overcast with a decidedly pale-brown tint thus *Givira cornelia* is not used in further comparisons. The forewings of *Givira durangona* are overcast with a gray tint from the post medial line to the outer margin (Fig. 7). The hindwings of *Givira durangona* are overcast with gray. The male genitalia of *Givira delindae*, *Givira carla*, and *Givira durangona* are closely similar in appearance. They are distinguished by subtle differences in shape and ratios of width to length of the valvae. The valvae of *Givira delindae* are 1.22× as long as wide, and they are not noticeably curved dorsad (Fig. 9). The valvae of *Givira durangona* are 1.25× as long as wide (Fig. 11), and they are slightly curved dorsad. The valvae of *Givira carla* are 1.78× as long as wide (Fig. 10), and in comparison, they are noticeably curved dorsad.

**Figures 13–15.**
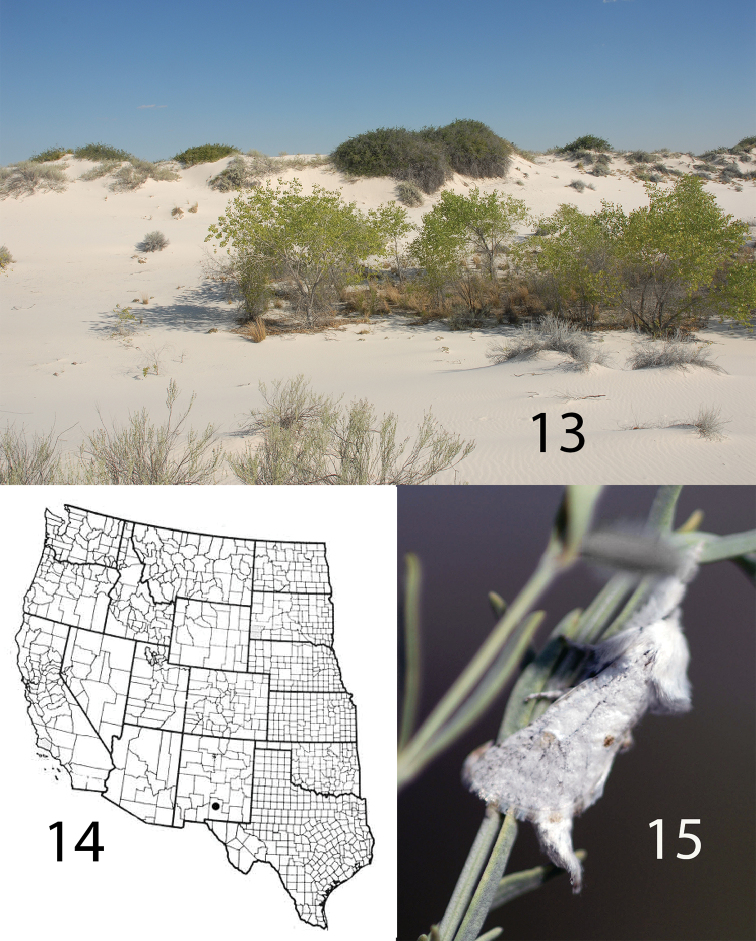
*Givira delindae* **13** type locality **14** distribution **15** Adult resting on a branch of frosted mint (*Poliomintha incana* (Torr.) A. Gray (Lamiaceae)), a common shrub in the dunes.

#### Description.

**Adult male** (Figs 1, 2): Head. Front and vertex smooth, scales directed forward and ventrad, white, narrowly spatulate, semi-erect; palpi short, extending to just dorsad of clypeus, straight, basal and middle segments equal length, apical segment 0.25× length of second segment, all three segments with semi-erect scales, gypsum-colored, long erect cactus-spine scales scattered on all surfaces. Haustellum obscured in dense scaling. Eyes naked, a few black hair-like long scales directed towards base of forewing from lateral posterior margin of eye. Antennae bipectinate, each ramus = 1.7× width of antennal shaft. Rami gradually shorter towards terminus; dorsal surface white scales, ventral surface naked, densely setose. Thorax. Thoracic scales hair-like, erect, fuzzy in appearance, white, tegulae similar; underside, scales hair-like, erect, fuzzy in appearance, concolorous. Fore legs denwsely scaled, white, lateral surfaces with long shaggy scales, scattered dark-gray scales, hair-like, erect, fuzzy appearance, hind leg femur one terminal pair of spurs. Forewing: length 9.6–16.2 mm, mean = 12.5 mm, n = 82, satiny white, triangular shaped, rounded apex, contrasting black patch on medial line near dorsal margin, a second dark patch may be present on post-medial line. Post-medial line with two patches erect scales, one subcoastal and one above dorsal margin, medial and post-medial lines pale-gray, not contrasting; underside white, postmedial and sub-terminal lines gray, dorsal margin white with 8–10 short contrasting perpendicular gray bars, terminal line gray, broken, fringe alternating gray and white patches; Hindwing faintly pale-gray, triangular, apex rounded, alternating white and gray lines parallel to outer margin, not contrasting, terminal line broken-gray patches, fringe white; underside white shaded with gray, terminal line dark gray, broken. Abdomen. Dorsum white, scales erect, fuzzy in appearance, two basal tufts with gray-tipped scales, furcate tuft on last segment with gray-tipped scales. Ventral scales white, erect, fuzzy in appearance. Male Genitalia (Figs 9, 9a), Uncus, apex slightly widened, blunt, curved ventrally; tegumen A shaped, width equals length; valvae straight, narrowed apically, apex rounded, curved mesially near apex, short, length = 1.22× width at base, setose, dense near apex, saccular region set off by a shallow depression at base of valve, costa turned 90° mesially and extended at base; juxta posterior part flat, jagged posterior margin, anterior part trough shaped, narrowed to blunt point; vinculum broad, robust, apex produced to truncated process with rounded corners. Aedeagus cylindrical, a rounded longitudinal keel-like structure at 2/5 length from anterior end, slightly bent at position of keel; anterior end gradually wider from bend to anterior opening; posterior end abruptly flared immediately before terminus (like the mouthpiece of a brass musical instrument). **Adult female** (Figs 3, 4): Habitus like male. Forewing: length 15.1–16.5 mm, mean = 15.7 mm, n = 3. Antennae bipectinate, each rhamus = 1 × width of antennal shaft. Rhami shorter towards terminal end of antennae. Abdomen. S-8 heavily sclerotized, lateral margins parallel, posterior margin deeply concave. Genitalia (Fig. 12). Papilla analis short, rubbery, retracted into abdomen, as wide as long, rounded, membranous, setose; posterior apophysis short, due to withdrawn papilla analis appears to be anterior of anterior apophysis, sinuous, extends to caudal end of concavity in S-8, terminal end spoon shaped; anterior apophysis slender, sinuous, with lateral processes, posterior end Y shaped, extends caudad of end of concavity in S-8, terminal end sinuous, spoon shaped; T-8 short, weakly sclerotized, translucent, posterior margin with numerous processes appearing like a comb with widely-spaced spine-like teeth. Ostium bursae, anterior margin a sclerotized ring, posterior margin lightly sclerotized, opens into a funnel-shaped sinus vaginalis. Ostium-bursae heavily sclerotized, protruding, ductus bursae lightly sclerotized, short, narrowed at midpoint, a sharp dogleg to juncture with appendix bursae and corpus bursae, appendix bursae round sclerotized, at right angle to juncture with ductus bursae and corpus bursae; corpus bursae round, short, flattened, appressed against S-8, dorsal surface rugose, sclerotized ridges a complex reticulated network, appearing cage-like.

#### Remarks.

This new species is placed in the genus *Givira* based on three character states as defined by Barnes and McDunnough (1911). 1) presence of a cross vein between A_1_ and A_2_ of the forewing (near the tornus in *Givira delindae*), 2) veins R and M_1_ of the hindwing stalked, and 3) one pair of apical spurs.

#### Biology and distribution.

*Givira delindae* occurs in White Sands National Monument, Otero County, New Mexico (Figs 13, 14). Several of the sample sites used for this study were not in the dunes. Only one specimen of *Givira delindae* was seen at any sample site outside the dunes. The single specimen captured outside the dunes was at an incandescent light 300 meters east of the dunes at the Administration Building. The immature stages and the larval host are unknown.

### Check list of the species of Cossidae recorded from White Sands National Monument

All were collected during this study (2007–2016)

Hypoptinae

*Givira* Walker, 1856

*cornelia* (Neumoegen & Dyar, 1893)

*delindae* Metzler, sp. n.

*durangona* (Schaus, 1901)

Cossinae

*Comadia* Barnes & McDunnough, 1911

*albistriga* (Barnes & McDunnough, 1918)

*henrici* (Grote, 1882)

*manfredi* (Neumoegen, 1884)

## Discussion

*Givira delindae*, *Givira carla*, and *Givira durangona* are closely similar in appearance. *Givira delindae* appears to be intermediate between *Givira carla* and *Givira durangona* both in maculation and male genital structure. *Givira delindae* came to my attention when I noticed that specimens of the small white Cossidae I collected had a satiny appearance of the forewings. I investigated further. The specimens quickly become greasy after which a positive identification is difficult. Identification is often not possible without examination of the male genitalia. All specimens should be degreased as matter of routine. I was able to detect that two of my specimens from White Sands are *Givira cornelia*, and two specimens are *Givira durangona* only after they were degreased.

The males and females of *Givira delindae* are essentially identical in appearance. The hindwings do not have frenulum acanthae. The slightly shorter antennal rhami (difficult to discern without magnification) of the females is the only outward clue to separate males from females. I found that if I carefully brushed away scales from the ventral surface of the apex of the abdomen using a blunted #000 or #0000 artist’s brush, I could see the ventral surface of the barely protruding valvae of the males. The scales can be gently brushed away without disturbing the furcate tuft of scales at the tip of the abdomen.

The internal structures of the female genitalia are arduous to dissect and even more difficult to discern because of the heavily sclerotized T-8 and fatty tissue in the abdomen. The short sclerotized ductus bursae allows very little tolerance to manipulate the structures without tearing the parts apart. The structures are nearly impossible to illustrate with photographs.

## Supplementary Material

XML Treatment for Givira delindae

## References

[B1] Barnes W McDunnough J (1911) Revision of the Cossidae of North America. Contributions to the Natural History of Lepidoptera of North America 1(1): 1–35.

[B2] Borth R Ivinskis P Saldaitis A Yakolev R (2011) Cossidae of Socotra Archipelago (Yemen). ZooKeys 122: 45–69. https://doi.org/10.3897/zookeys.122.1213 10.3897/zookeys.122.1213 PMC3187673 21998527

[B3] Clarke JFG (1941) The preparation of slides of the genitalia of Lepidoptera. Bulletin of the Brooklyn Entomological Society 36: 149–161.

[B4] Clench HK (1956) New Neotropical Cossidae (Lepidoptera). Annals and Magazine of Natural History 12(9)[1957]: 897–906. https://doi.org/10.1080/00222935608655914

[B5] Clench HK (1957) Cossidae from Chile (Lepidoptera). Mitteilungen der Münchner Entomologischen Gesellschaft 47: 122–142.

[B6] Covell CV Jr (1984) A Field Guide to the Moths of Eastern North America. [Peterson Field Guide Saeries]. Houghton Mifflin Company, Boston, Massachusetts, 496 pp.

[B7] Dalle Torre KW von (1923) Cossidae. In: Strand E (Ed.) Lepidopterorum Catalogus, pars 29. W. Junk, Berlin, 1–63.

[B8] Daniel F (1956) Monographie der Cossidae II. Die genera *Cossus* Fabr. und *Lamellacossus* gen. N. (Lep). Mitteilungen der Münchner Entomologischen Gesellschaft 44/45: 243–286.

[B9] Daniel F (1958) Monographie der Cossidae III. Das genus *Holcocerus* Stgr. Mitteilungen der Münchner Entomologischen Gesellschaft 49: 102–160.

[B10] Daniel F (1960) Monographie der Cossidae IV. Die genera *Cossulinus* Kby., *Dyspessacoccus* Dan. und *Isoceras* Tti. (Lep.). Mitteilungen der Münchner Entomologischen Gesellschaft 50: 93–118.

[B11] Daniel F (1962) Monographie der Cossidae VI. Genus *Dyspessa* Hbn. Erster Teil. Mitteilungen der Münchner Entomologischen Gesellschaft 52: 1–38.

[B12] Daniel F (1964) Monographie der Cossidae VII. Genus *Dyspessa* Hbn. Zweiter Teil Genus *Paropta* Stgr. Mitteilungen der Münchner Entomologischen Gesellschaft 54: 181–236.

[B13] Daniel F (1965) Monographie der Cossidae VIII. Nachtrage und register zur subfamilie Cossinae. Mitteilungen der Münchner Entomologischen Gesellschaft 55: 77–114.

[B14] Donahue JP (1995) Cossidae. In: Heppner JB (Ed.) Atlas of Neotropical Lepidoptera Volume 3 Checklist: Part 2 Hyblaeoidea – Pyraloidea – Tortricoidea. Scientific Publishers, Gainesville, 122–127.

[B15] Dyar HG Schaus W (1937) Family: Cossidae. In: Seitz A (Ed.) The Macrolepidoptera of the World Vol. 6, The American Bombyces and Sphinges. Alfred Kernen, Publisher, Stuttgart, 1264–1287.

[B16] Edwards ED Gentili P Horak M Kristensen NP Nielsen ES (1998) The Cossoid/Sesioid Assemblage, Chapter 11. In: Kristensen NP (Ed.) Handbook of Zoology Part 35 Lepidopera, Moths and Butterflies Volume 1: Evolution, Systematics, and Biogeography. Walter de Gruyter, Berlin, 181–197.

[B17] Hardwick DF (1950) Preparation of slide mounts of lepidopterous genitalia. The Canadian Entomologist 82: 231–235. https://doi.org/10.4039/Ent82231-11

[B18] Hodges RW (1983) Cossidae. In: Hodges RW Dominick T Davis DR Ferguson DC Franclemont JG Munroe E Powell JA (Eds) Check List of the Lepidoptera of America North of Mexico including Greenland. E.W. Classey and Wedge Entomological Research Foundation, London, 30–31.

[B19] Hua BZ (2001(2002)) Cossidae. In: Huang BK (Ed.) Fauna of Insects in Fujian Province of China Vol. 5. Fujian Science and Technology Publishing House, Fuzhou, 6–10. [In Chinese]

[B20] Hua BZ Chou I Fang DQ Chen SL (1990) The Cossid Fauna of China (Lepidoptera: Cossidae. Tianzxe Eldonejo, Yanglibng, Shaanxi, 148 pp. [In Chinese]

[B21] Jewell EJ Abate F (2001) (Eds) The New Oxford American Dictionary. Oxford University Press, New York, 2023 pp.

[B22] Jimbo U (2011) Cossidae. In: Komaki F Koshiyasu Y Nasu Y Saito T (Eds) A Guide to the Lepidoptera of Japan. Tokai University Press, Kanagawa, 266–270. [In Japanese]

[B23] Klots AB (1970) Lepidoptera. In: Tuxen SL (Ed.) Taxonomist’s Glossary of Genitalia in Insects. Second Enlarged Edition, Chapter 20. S-H Service Agency, Inc., Darien, 115–130.

[B24] Lafontaine JD (2004) The Moths of North America Including Greenland, Fascicle 27.1, NoctuoideaNoctuidae (part) Noctuinae (part -Agrotini). The Wedge Entomological Research Foundation, Washington, DC, 385 pp.

[B25] Metzler EH (2014) The Lepidoptera of White Sands National Monument 6: A new species of *Chionodes* Hübner, [1825] (Lepidoptera, Gelechiidae, Gelechiinae) dedicated to Ronald W. Hodges and Elaine R. Snyder Hodges in the year of Ron’s 80th birthday. Journal of The Lepidopterists’ Society 68: 80–84. https://doi.org/10.18473/lepi.v68i2.a2

[B26] Metzler EH (2016) The Lepidoptera of White Sands National Monument, Otero County, New Mexico, USA 11. A new species of *Arotrura* Walsingham 1888 (Scythrididae), another iconic species from the Monument. Journal of The Lepidopterists’ Society 70: 194–200. https://doi.org/10.18473/107.070.0304

[B27] Metzler EH Bustos D Forbes GS (2009) The Lepidoptera of White Sands National Monument, Otero County, New Mexico, USA 1. Two new species of Noctuidae (Lepidoptera, Noctuinae, Agrotini). ZooKeys 9: 47–62. https://doi.org/10.3897/zookeys.9.182

[B28] Metzler EH Forbes GS (2011a) The Lepidoptera of White Sands National Monument, Otero County, New Mexico, USA 3. A new species of *Aleptina* Dyar, 1902 (Lepidoptera, Noctuidae, Amphipyrinae, Psaphidini). In: Schmidt BC Lafontaine JD (Eds) Contributions to the systematics of New World macro-moths III. ZooKeys 149: 125–133. 10.3897/zookeys.149.1517 PMC3234415 22207800

[B29] Metzler EH Forbes GS (2011b) The Lepidoptera of White Sands National Monument, Otero County, New Mexico, USA 4. A new species of *Schinia* Hübner, 1818 (Lepidoptera, Noctuidae, Heliothinae). In: Schmidt BC Lafontaine JD (Eds) Contributions to the systematics of New World macro-moths III. ZooKeys 149: 135–144. 10.3897/zookeys.149.1518 PMC3234416 22207801

[B30] Metzler EH Forbes GS (2012) The Lepidoptera of White Sands National Monument 5: Two new species of Cochylini (Lepidoptera, Tortricidae, Tortricinae). ZooTaxa 3444: 51–60.

[B31] Metzler EH Forbes GS Bustos D West R (2010) First records, representing major range extensions, of three species of Lepidoptera (Erebidae, Noctuidae, and Lasiocampidae) from New Mexico. Southwestern Entomologist 35(3): 309–311. https://doi.org/10.3958/059.035.0309

[B32] Metzler EH Landry JF (2016) The Lepidoptera of White Sands National Monument, Otero County, New Mexico, USA 10. A remarkable new white species of *Chionodes* Hübner (Gelechiidae). Zootaxa 4109: 372–380. https://doi.org/10.11646/zootaxa.4109.3.7 27394871 10.11646/zootaxa.4109.3.7

[B33] Metzler EH Lightfoot DC (2014) The Lepidoptera of White Sands National Monument 7: A new species of the genus *Areniscythris* (Scythrididae), a recently discovered iconic species from the Monument. Journal of The Lepidopterists’ Society 68(3): 185–190.

[B34] Mikkola K Lafontaine JD Gill JD (2009) The Moths of North America Fascicle 26.9 NoctuoideaNoctuidae (Part) Xyleninae (Part) Apameini (Part – Apamea group of genera). The Wedge Entomological Research Foundation, Washington, DC, 192 pp.

[B35] Pogue MG (2002) A world revision of the genus *Spodoptera* Guenée (Lepidoptera: Noctuidae). Memoirs of the American Entomology Society 43: 1–202.

[B36] Roepke W (1957) The cossids of the Malay region (Lepidoptera: Heterocera). Verhandelingen der koninklijke Nederlandse Akademie van Wetenschappen, Afd. Natuurkunde 52(1): 1–60.

[B37] Saldaitis A Ivinskis P (2010a) *Meharia yakovlevi*, a new species (Lepidoptera, Cossidae) from Yemen. Esperiana 15: 379–381.

[B38] Saldaitis A Ivinskis P (2010b) *Wittocossus dellabrunai* (Lepidoptera, Cossidae), a new species from China. Esperiana 15: 383–385.

[B39] Schneider-Hector D (1993) White Sands The History of a National Monument. The University of New Mexico Press. Albuquerque, New Mexico, 270 pp.

[B40] Schoorl JW (1990) A phylogenetic study on Cossidae (Lepidoptera: Ditrysia) based on external adult morphology. Zoologische Verhandelingen 263: 1–295.

[B41] Smith JS Jr Stanley JM Hartsock JG Campbell LW (1974) S-1 Black-light Insect-survey Trap Plans and Specifications. ARS-S-31. New Orleans, 4 pp.

[B42] Stroud CP (1950) A survey of the insects of White Sands National Monument, Tularosa Basin, New Mexico. American Midland Naturalist 44(3): 659–677. https://doi.org/10.2307/2421827

[B43] Ureta E (1957) Revision de la familia Cossidae (Lep. Het.) en Chile. Boletin del Museo Nacional de Historia Natural 27(2): 129–153.

[B44] Wiltshire E (1982) Insects of Saudi Arabia Lepidoptera: Fam. Cossidae, Zygaenidae, Sesiidae, Lasiocampidae, Bombycidae, Sphingidae, Thaumetopoeidae, Thyretidae, Notodontidae, Geometridae, Lymantriidae, Noctuidae, Ctenuchidae (Part 2). In: Wittmer W Büttiker W (Eds) Fauna of Saudi Arabia Vol 4 271–332.

[B45] Wright DJ (2012) Eight new species of *Eucosma* Hübner (Tortricidae) from western North America. Journal of The Lepidopterists’ Society 66(1): 27–40. https://doi.org/10.18473/lepi.v66i1.a3

[B46] Wright DJ (2014) Four new *Eucosma* (Tortricidae) from southwestern United States. Journal of The Lepidopterists’ Society 68(3): 191–196. https://doi.org/10.18473/lepi.v68i3.a6

[B47] Wright DJ Gilligan TM (2015) *Eucosma* Hübner of the contiguous United States and Canada (Lepidoptera: Tortricidae: Eucosmini). Wedge Entomological Research Foundation, Alamogordo, 255 pp. [31 col. plates]

[B48] Yakovlev RV (2008a) New species of Palaearctic and Oriental Cossidae (Lepidoptera). I. Two new species from genus *Cossulus* Staudinger, 1887 form Turkey and Kyrgystan. Eversmannia 15/16: 44–47.

[B49] Yakovlev RV (2008b) New species of Palaearctic and Oriental Cossidae (Lepidoptera). IV. New taxa of the genus *Dyspessa* Hübner, [1820] from south-western Palaearctic. Eversmannia 15/16: 53–68.

[B50] Yakovlev RV (2011a) Two new species of the goat moths (Lepidoptera, Cossidae) from New Guinea. Amurian Zoological Journal 3(3): 284–286.

[B51] Yakovlev RV (2011b) A new subfamily of Palaearctic carpenter-moths (Lepidoptera, Cossidae). Entomological Review 90(1): 216–222. [In Russian]

[B52] Yakovlev RV (2011c) A brief review of the genus *Cecryphalus* Schoorl, 1990 (Lepidoptera: Cossidae). Eurasian Entomological Journal 10(1): 19–21.

[B53] Yakovlev RV (2015a) A new species of *Isocossus* Roepke, 1957 (Lepidoptera: Cossidae) from Vietnam, including a world catalogue of the genus. Zootaxa 3990: 141–146. https://doi.org/10.11646/zootaxa.3990.1.9 26250225 10.11646/zootaxa.3990.1.9

[B54] Yakovlev RV (2015b) A new species of *Isoceras* Turati, 1924 (Lepidoptera: Cossidae) from Armenia. Zootaxa 3990: 147–150. https://doi.org/10.11646/zootaxa.3990.1.10 26250226 10.11646/zootaxa.3990.1.10

[B55] Yakovlev RV Saldaitis A (2008) New species of Palaearctic and Oriental Cossidae (Lepidoptera) III. New species of genus *Meharia* Chretien, 1915 from Morocco. Eversmannia 15/16: 49–52.

[B56] Yakovlev RV Ivinskis P Rimsaite J Saldaitis A (2013) Description of two new species of *Meharia* Chretien, 1915 (Lepidoptera: Cossidae) from east Africa. Tinea 20(2): 105–107. https://doi.org/10.11646/zootaxa.3635.5.9 10.11646/zootaxa.3635.5.9 26097971

[B57] Yakovlev RV Poltavsky AN Ilyina EV (2015) Cossidae (Lepidoptera) of the Russian Caucasus with the description of a new species. Zootaxa 4044: 270–288. https://doi.org/10.11646/zootaxa.4044.2.5 26624712 10.11646/zootaxa.4044.2.5

[B58] Yakovlev RV Witt TJ (2015) *Orientozeuzera martinii* sp. nov., a new species of Carpenter-Moths (Lepidoptera: Cossidae) from Borneo. Zootaxa 3990: 138–140. https://doi.org/10.11646/zootaxa.3990.1.8 26250224 10.11646/zootaxa.3990.1.8

[B59] Yakovlev RV Witt TJ (2016) A world catalogue of *Phragmataecia* (Lepidoptera: Cossidae), with a new species from Kazakhstan and Kyrgyzstan. Zootaxa 4085: 589–600. https://doi.org/10.11646/zootaxa.4085.4.8 27394321 10.11646/zootaxa.4085.4.8

[B60] Zagulajev AK (1978) Family Cossidae—Carpenter Moths In: Medvedev GS (Ed.) Keys to the Insects of the European part of the USSR IV, Part One. Nauka Publishers, Leningrad, 177–186. [In Russian]

[B61] Zukowsky B (1954) Aegeriidae, Cossidae, und Hepialidae (Lep.). In: Titschack E (Ed.) Beitrage zur Fauna Perus. Band IV. Verlag von Gustav Fischer, Jena, 85–94.

